# Supplementary material for: Multispecies biofilm architecture determines bacterial exposure to phages
Source: PLoS Biol. 2022 Dec 22;20(12):e3001913. doi: 10.1371/journal.pbio.3001913 (PMC9778933; doi:10.1371/journal.pbio.3001913)
Supplement: S1 Fig — (A) A co-culture biofilm of V. cholerae (purple) and E. coli (yellow) after 16 h of continuous phage exposure. (B) The same microcolony as (A) after heavy disturbance to clear E. coli cells out of the chambers to test for phage resistance. (C) E. coli CFU recovered from co-coculture flow devices when plated without T7 phages (for total counts) or plates saturated with T7 phages (for de novo T7-resistant mutants) (n = 4). (D) E. coli CFU in liquid culture with V. cholerae over time with and without the addition of phages. The addition of V. cholerae in shaken liquid culture did not confer protection against phage exposure (n = 3). The data underlying this figure can be found in S1 Data. (PDF) [file pbio.3001913.s003.pdf]

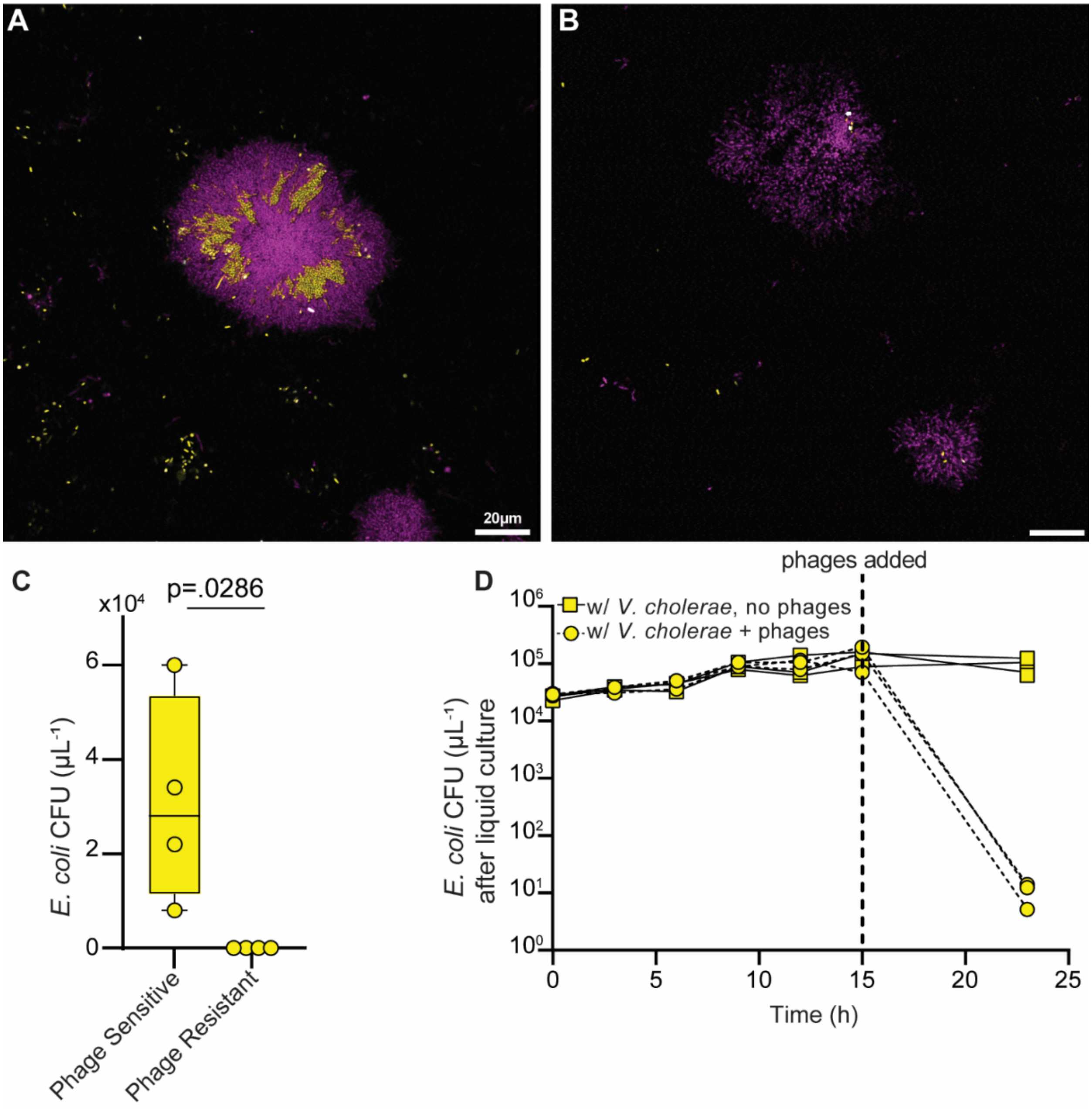

SI Figure S1. *E. coli* cells can survive T7 phage exposure within multispecies biofilms in the absence of *de novo* phage resistance evolution. (**A**) A co-culture biofilm of *V. cholerae* (purple) and *E. coli* (yellow) after 16 h of continuous phage exposure. (**B**) The same microcolony as (**A**) after heavy disturbance to clear *E. coli* cells out of the chambers to test for phage resistance. (**C**) *E. coli* CFU recovered from co-coculture flow devices when plated without T7 phages (for total counts) or plates saturated with T7 phages (for *de novo* T7-resistant mutants) (*n*=4). (**D**) *E. coli* CFU in liquid culture with *V. cholerae* over time with and without the addition of phages. The addition of *V. cholerae* in shaken liquid culture did not confer protection against phage exposure (*n*=3). The data underlying this figure can be found in S1 Data.
