# Supplementary material for: Multispecies biofilm architecture determines bacterial exposure to phages
Source: PLoS Biol. 2022 Dec 22;20(12):e3001913. doi: 10.1371/journal.pbio.3001913 (PMC9778933; doi:10.1371/journal.pbio.3001913)
Supplement: S2 Fig — (A, B) We surveyed biofilms extensively to see if E. coli ever survived when phages were added from the beginning of biofilm growth. Sporadic E. coli that had survived phage exposure could be found, but only very rarely. The image in panel (A) is one of only 3 instances out of hundreds of images in which any E. coli were found. (C) E. coli total abundance over time when phages are added continuously from the beginning of biofilm growth either in monoculture or with V. cholerae (n = 6–12). The data underlying this figure can be found in S1 Data. (PDF) [file pbio.3001913.s004.pdf]

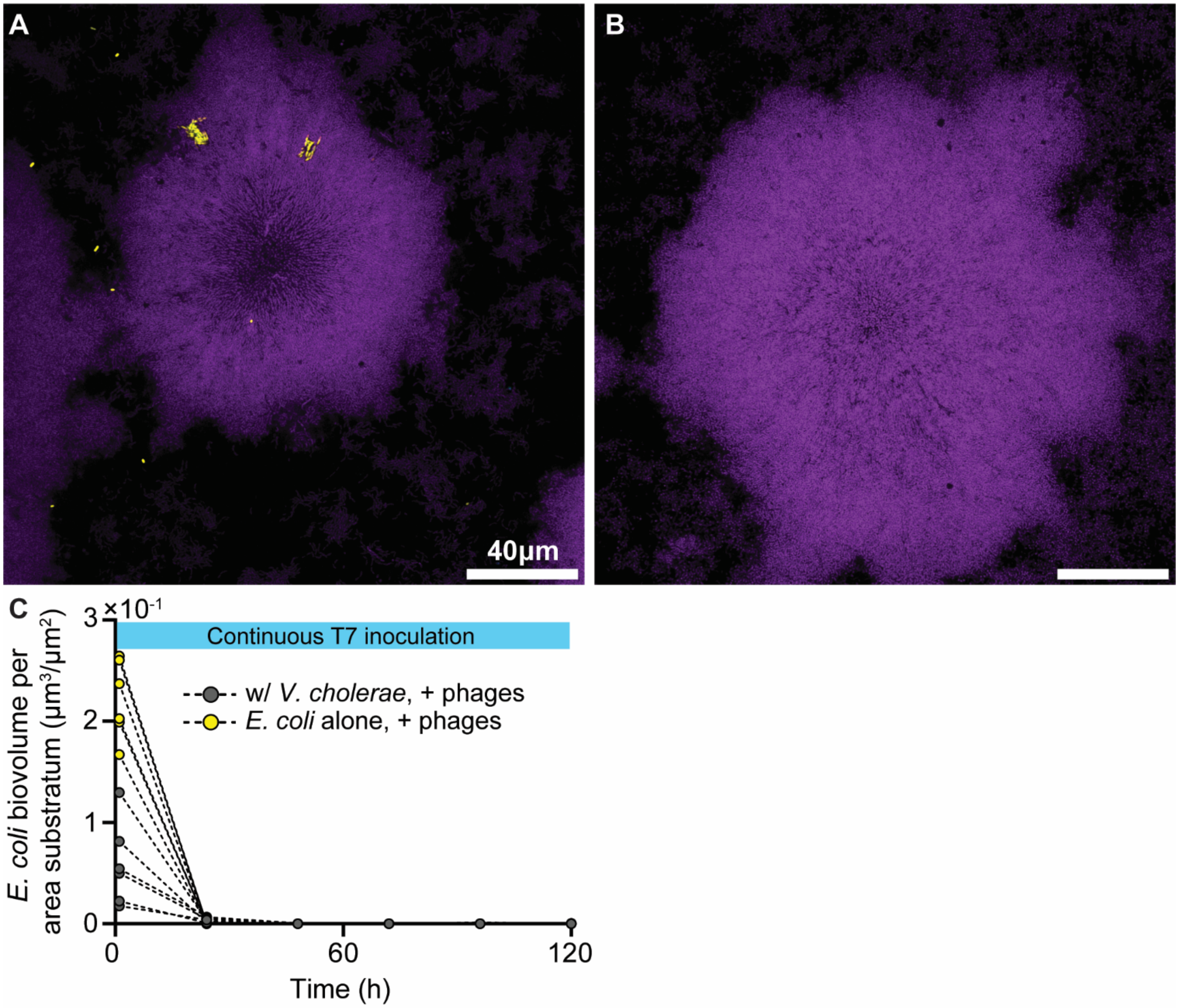

SI Figure S2. *E. coli* (yellow) does not gain phage exposure protection in co-culture with *V. cholerae* (purple) if phages are added to the culture from the beginning of biofilm growth. (**A,B**) We surveyed biofilms extensively to see if *E. coli* ever survived when phages were added from the beginning of biofilm growth. Sporadic *E. coli* that had survived phage exposure could be found, but only very rarely. The image in panel (A) is one of only 3 instances out of hundreds of images in which any *E. coli* were found. (**C**) *E. coli* total abundance over time when phages are added continuously from the beginning of biofilm growth either in monoculture or with *V. cholerae* (*n*=6-12). The data underlying this figure can be found in S1 Data.
