# Supplementary material for: Multispecies biofilm architecture determines bacterial exposure to phages
Source: PLoS Biol. 2022 Dec 22;20(12):e3001913. doi: 10.1371/journal.pbio.3001913 (PMC9778933; doi:10.1371/journal.pbio.3001913)
Supplement: S3 Fig — (A) 3D rendering of V. cholerae (purple), E. coli (yellow), and E. coli with λ phages attached to their cell surface (red). (B) Quantification of E. coli and λ phage overlap in a top-down view of the biofilm rendered in panel A. E. coli clusters within V. cholerae biofilms generally evade λ phages, as seen with T7 phages. (C) Frequency of phage infection, measured by Mander’s overlap coefficient between E. coli and λ phage fluorescent signal, as a function of the V. cholerae fluorescence shell in proximity to E. coli. The data underlying this figure can be found in S1 Data. (PDF) [file pbio.3001913.s005.pdf]

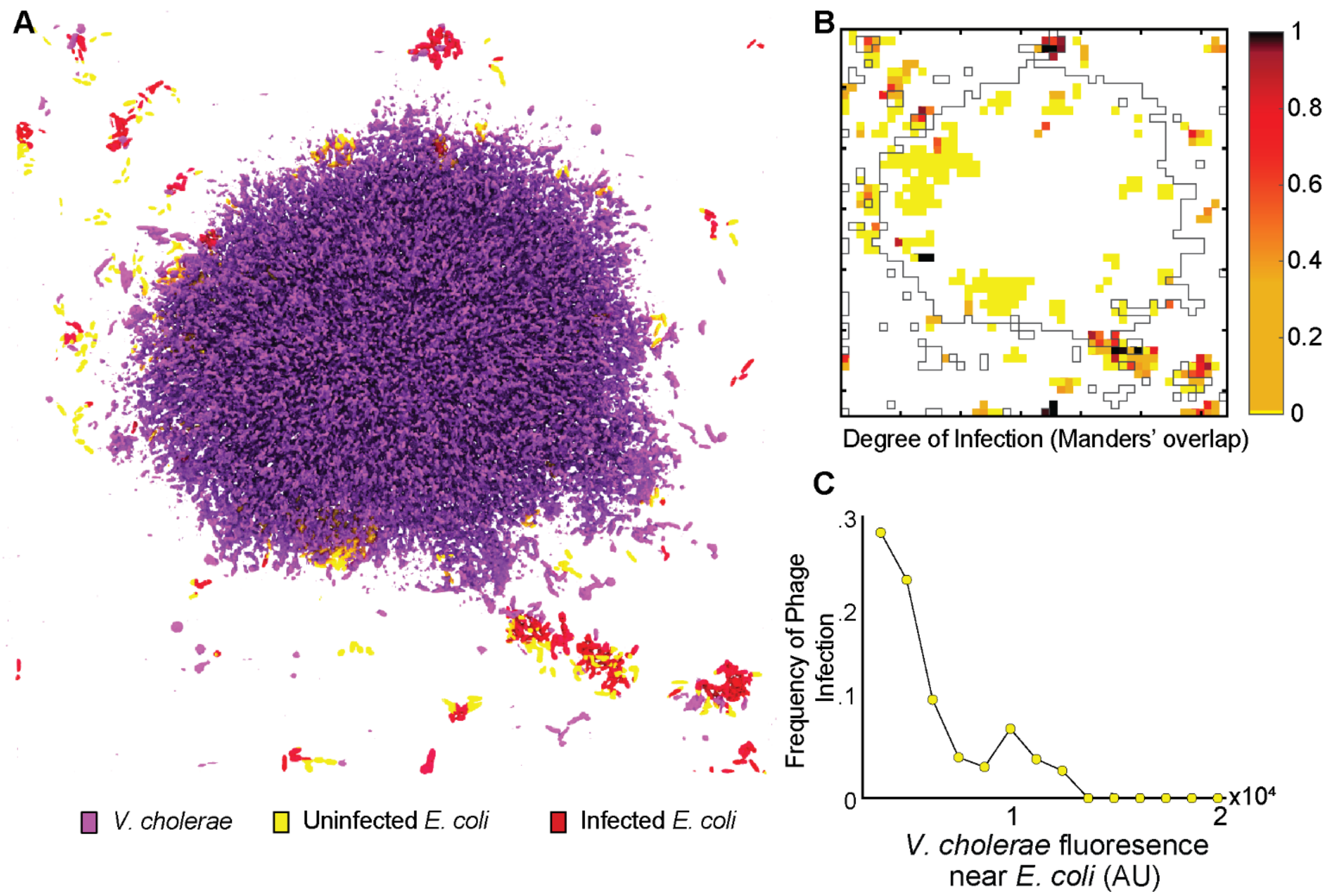

SI Figure S3. *E. coli* cells can evade exposure to λ phages when embedded in *V. cholerae* cell groups in the same manner as observed for T7 phage exposure. (**A**) 3D rendering of *V. cholerae* (purple), *E. coli* (yellow), and *E. coli* with λ phages attached to their cell surface (red). (**B**) Quantification of *E. coli* and λ phage overlap in a top-down view of the biofilm rendered in panel A. *E. coli* clusters within *V. cholerae* biofilms generally evade λ phages, as seen with T7 phages. (**C**) Frequency of phage infection, measured by Mander's overlap coefficient between *E. coli* and λ phage fluorescent signal, as a function of the *V. cholerae* fluorescence shell in proximity to *E. coli*. The data underlying this figure can be found in S1 Data.
