# Supplementary material for: Multispecies biofilm architecture determines bacterial exposure to phages
Source: PLoS Biol. 2022 Dec 22;20(12):e3001913. doi: 10.1371/journal.pbio.3001913 (PMC9778933; doi:10.1371/journal.pbio.3001913)
Supplement: S4 Fig — (A) Representative image of a triculture condition of ΔrbmA V. cholerae, V. cholerae, and E. coli. (B) The neighborhood biovolume fraction of the merged biovolumes of both V. cholerae genotypes and E. coli from (A). The data underlying this figure can be found in S1 Data. (PDF) [file pbio.3001913.s006.pdf]

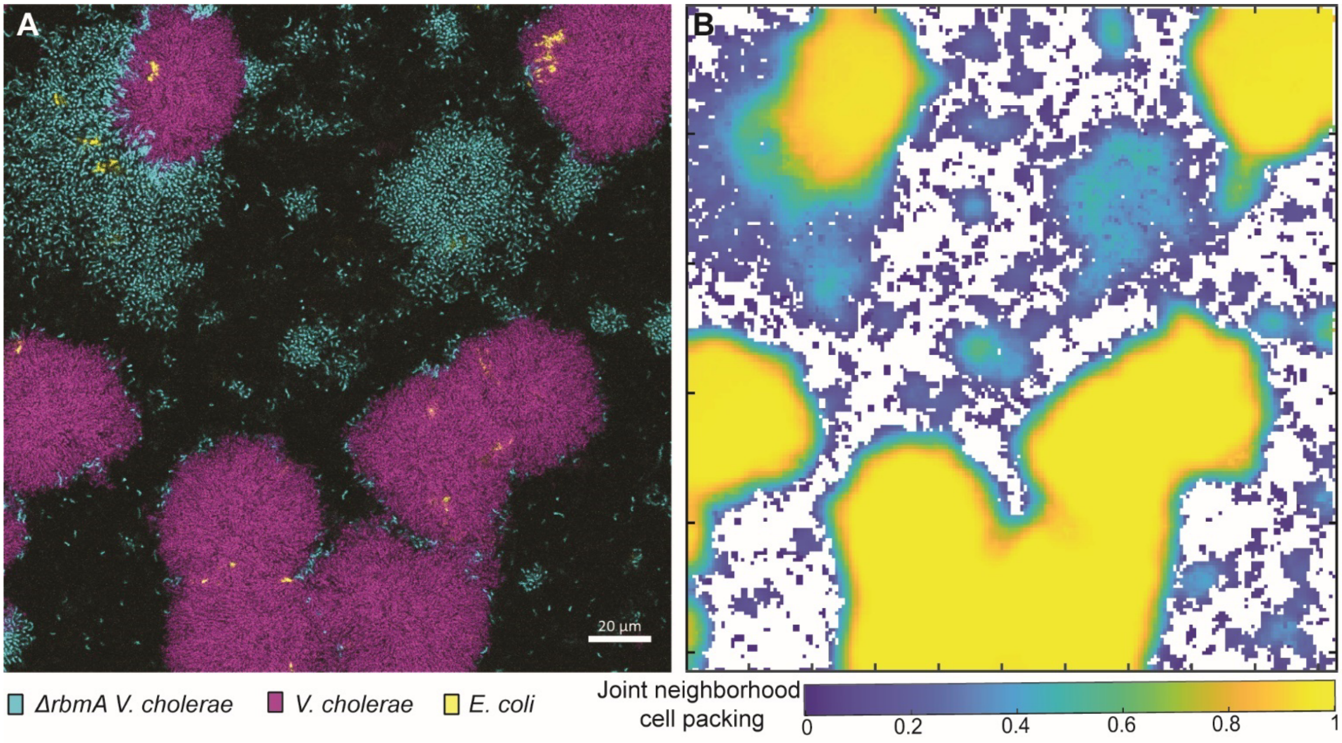

SI Figure S4. Quantification of cell packing for WT *V. cholerae* (purple) and ∆*rbmA V. cholerae* (cyan) in co-culture with *E. coli* (yellow). (**A**) Representative image of a triculture condition of ∆*rbmA V. cholerae, V. cholerae,* and *E. coli*. (**B**) The neighborhood biovolume fraction of the merged biovolumes of both *V. cholerae* genotypes and *E. coli* from (**A**). The data underlying this figure can be found in S1 Data.
