# Supplementary material for: Multispecies biofilm architecture determines bacterial exposure to phages
Source: PLoS Biol. 2022 Dec 22;20(12):e3001913. doi: 10.1371/journal.pbio.3001913 (PMC9778933; doi:10.1371/journal.pbio.3001913)
Supplement: S5 Fig — (PDF) [file pbio.3001913.s007.pdf]

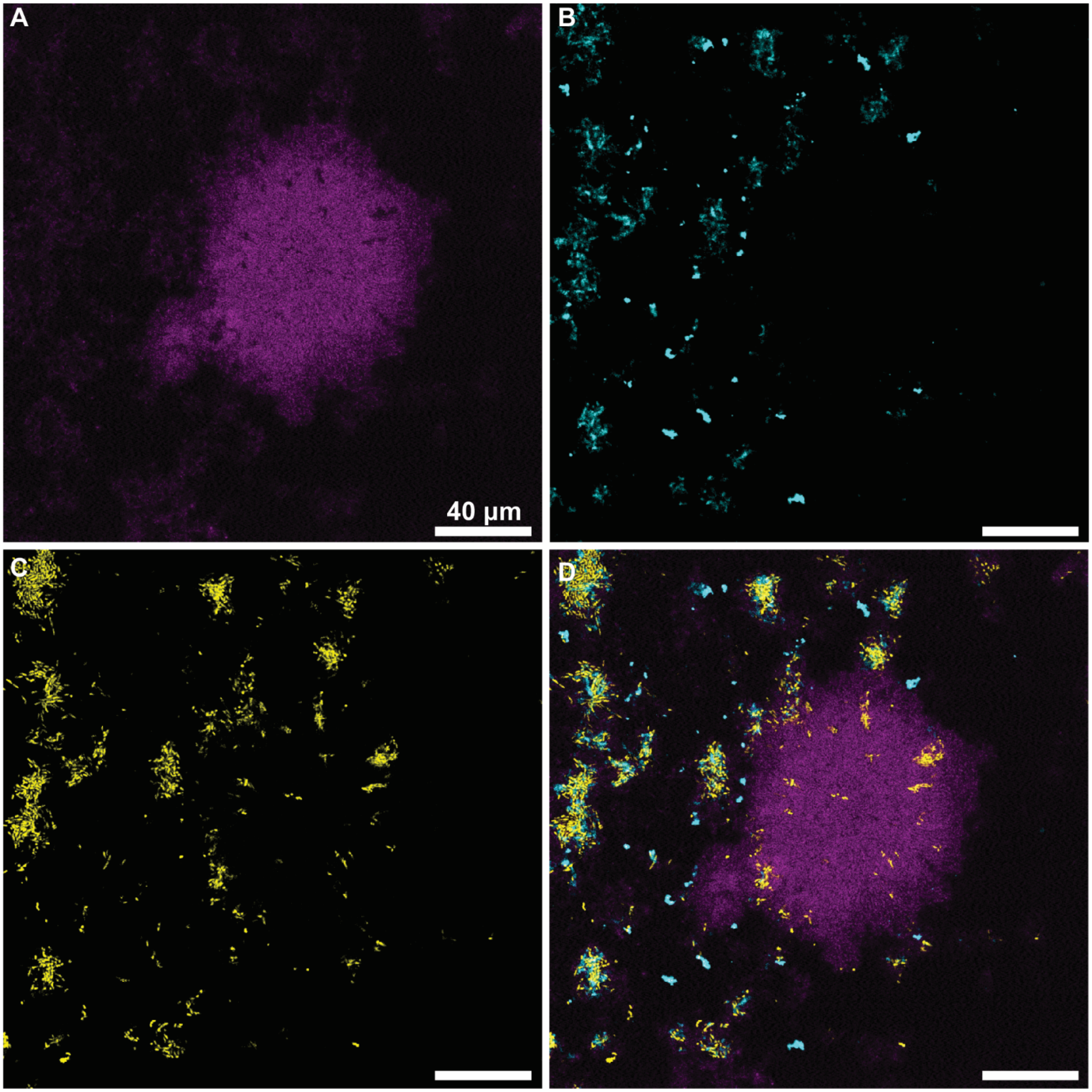

SI Figure S5. Co-culture biofilms exposed to dye-conjugated T7 phages (cyan) show minimal association of phages to *V. cholerae* cell groups (purple) and high T7 localization to *E. coli* (yellow).
