# Supplementary material for: Multispecies biofilm architecture determines bacterial exposure to phages
Source: PLoS Biol. 2022 Dec 22;20(12):e3001913. doi: 10.1371/journal.pbio.3001913 (PMC9778933; doi:10.1371/journal.pbio.3001913)
Supplement: S6 Fig — V. cholerae biofilms (purple) grown while dye-conjugated T7 phages (cyan) were continously added into the flow devices from the beginning of biofilm growth for 96 h. (A–D) Representative image slices taken from a biofilm (A) 1.54 μm, (B) 2.70 μm, (C) 4.25 μm, and (D) 13.90 μm above the glass, respectively. The restriction of phages to the bottom layer of the V. cholerae biofilm most likely indicates that these phages initially attached to the underlying glass surface and were overgrown by the expanding V. cholerae cell group as it expanded from its initial position of attachment. (PDF) [file pbio.3001913.s008.pdf]

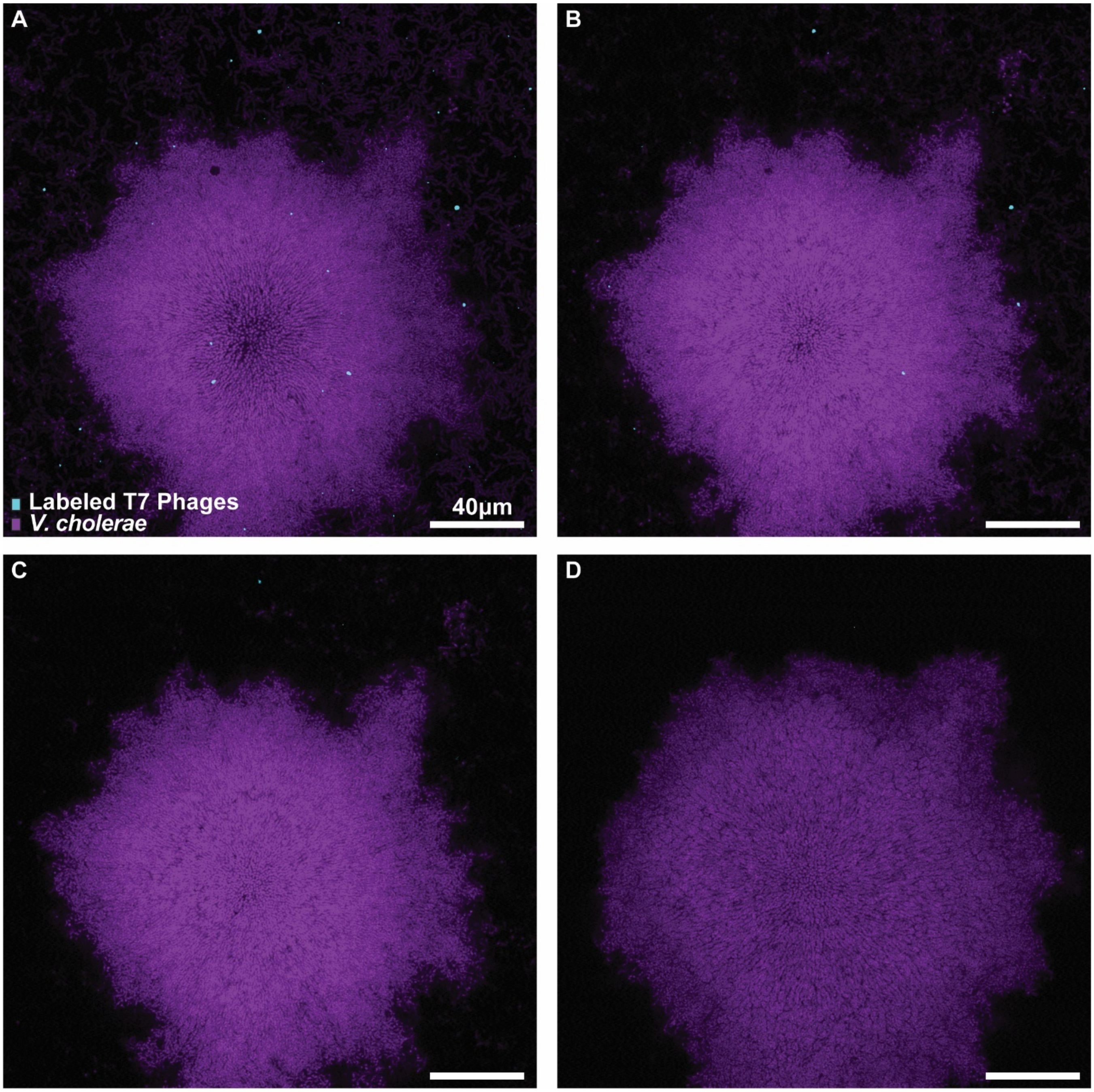

SI Figure S6. T7 phages do not generally enter the interior or accumulate on the outer periphery of *V. cholerae* biofilms. *V. cholerae* biofilms (purple) grown while dye-conjugated T7 phages (cyan) were continously added into the flow devices from the beginning of biofilm growth for 96 h. (**A-D**) Representative image slices taken from a biofilm (A) 1.54μm, (B) 2.70μm, (C) 4.25μm, and (D) 13.90μm above the glass, respectively. The restriction of phages to the bottom layer of the *V. cholerae* biofilm most likely indicates that these phages initially attached to the underlying glass surface and were over-grown by the expanding *V. cholerae* cell group as it expanded from its initial position of attachment.
