# Supplementary material for: Multispecies biofilm architecture determines bacterial exposure to phages
Source: PLoS Biol. 2022 Dec 22;20(12):e3001913. doi: 10.1371/journal.pbio.3001913 (PMC9778933; doi:10.1371/journal.pbio.3001913)
Supplement: S7 Fig — (A) csgBAC transcription as a function of distance of E. coli cells from the nearest V. cholerae. The data underlying this figure can be found in S1 Data. (PDF) [file pbio.3001913.s009.pdf]

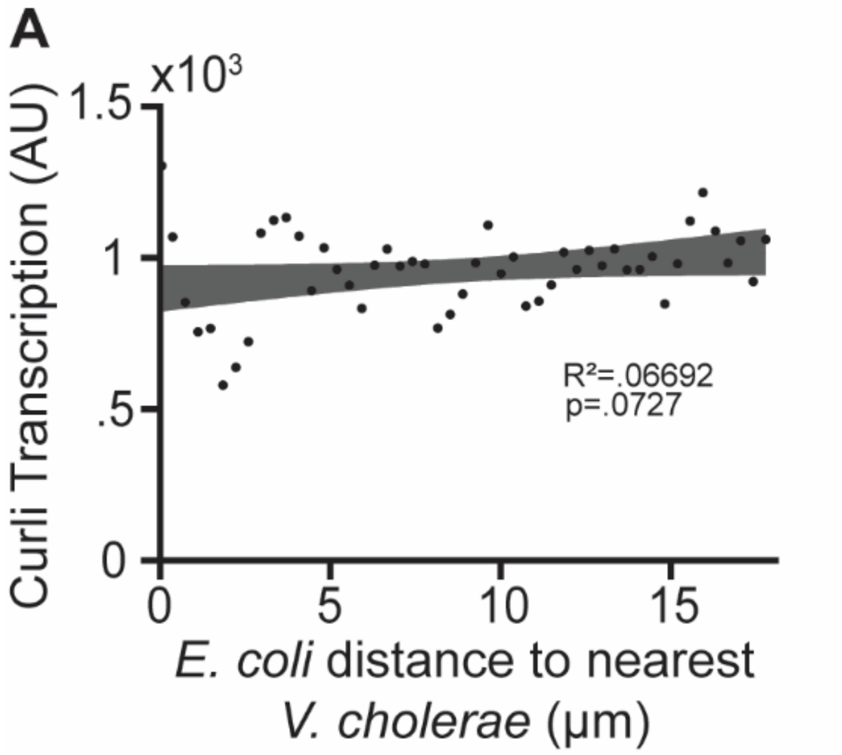

SI Figure S7. Within co-culture flow devices, *E. coli* cells exhibit similar levels *csgBAC* transcription independently of their distance from *V. cholerae*. (**A**) *csgBAC* transcription as a function of distance of *E. coli* cells from the nearest *V. cholerae*. The data underlying this figure can be found in S1 Data.
