# Supplementary material for: Multispecies biofilm architecture determines bacterial exposure to phages
Source: PLoS Biol. 2022 Dec 22;20(12):e3001913. doi: 10.1371/journal.pbio.3001913 (PMC9778933; doi:10.1371/journal.pbio.3001913)
Supplement: S8 Fig — (A) E. coli frequency in biofilm co-culture with V. cholerae, with and without the introduction of phages (n = 4). (B) V. cholerae absolute abundance in monoculture and in co-culture with E. coli after 120 h of biofilm growth (Mann–Whitney U test with n = 8, n = 16). The data underlying this figure can be found in S1 Data. (PDF) [file pbio.3001913.s010.pdf]

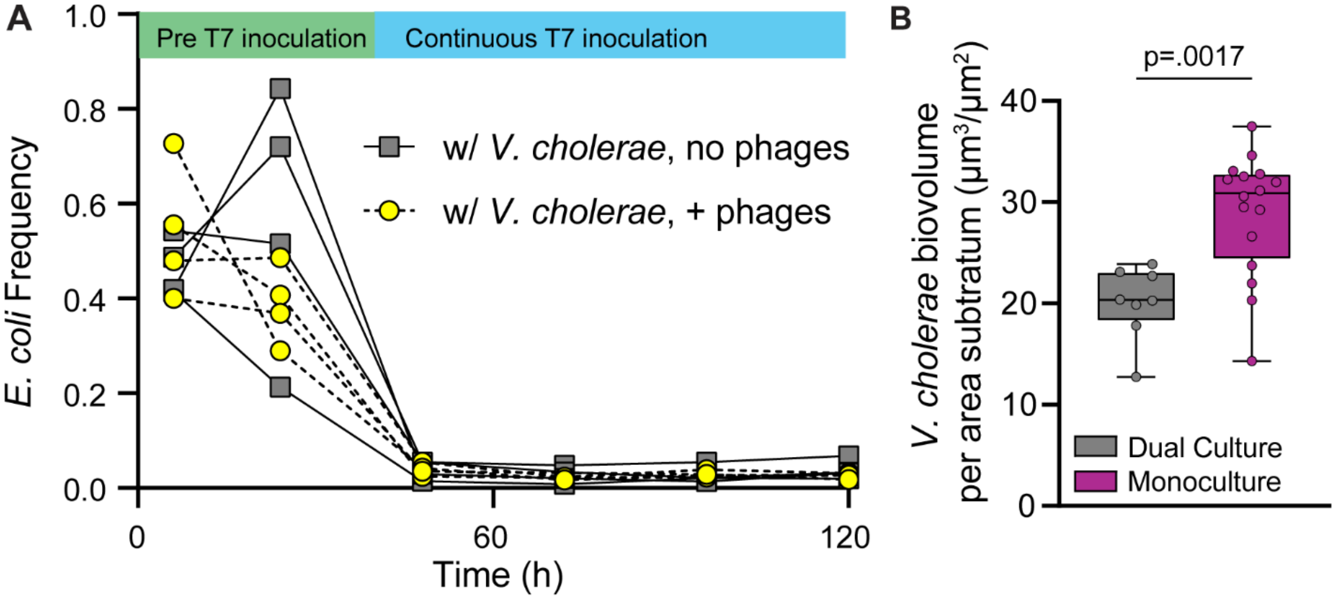

SI Figure S8. *E. coli* and *V. cholerae* compete for space and nutrients with *E. coli* falling to a steady state frequency of 2-5% from a range of different starting frequencies. (**A**) *E. coli* frequency in biofilm co-culture with *V. cholerae*, with and without the introduction of phages (*n*=4). (**B**) *V. cholerae* absolute abundance in monoculture and in co-culture with *E. coli* after 120 h of biofilm growth. (Mann-Whitney U-test with *n*=8, *n*=16). The data underlying this figure can be found in S1 Data.
