# Supplementary material for: Multispecies biofilm architecture determines bacterial exposure to phages
Source: PLoS Biol. 2022 Dec 22;20(12):e3001913. doi: 10.1371/journal.pbio.3001913 (PMC9778933; doi:10.1371/journal.pbio.3001913)
Supplement: S9 Fig — (A) Heatmaps of the merged neighborhood biovolume fraction for both cell types over the time course experiment shown in (B). (B) Time course of a dual culture biofilm of V. cholerae (purple) and E. coli (yellow), with phages being introduced into this system from 48 h onward. (C) Time course of a monoculture biofilm of E. coli, with phages introduced from 48 h onward. (D) Heatmaps of the merged neighborhood biovolume fraction for the time course shown in (D). The data underlying this figure can be found in S1 Data. (PDF) [file pbio.3001913.s011.pdf]

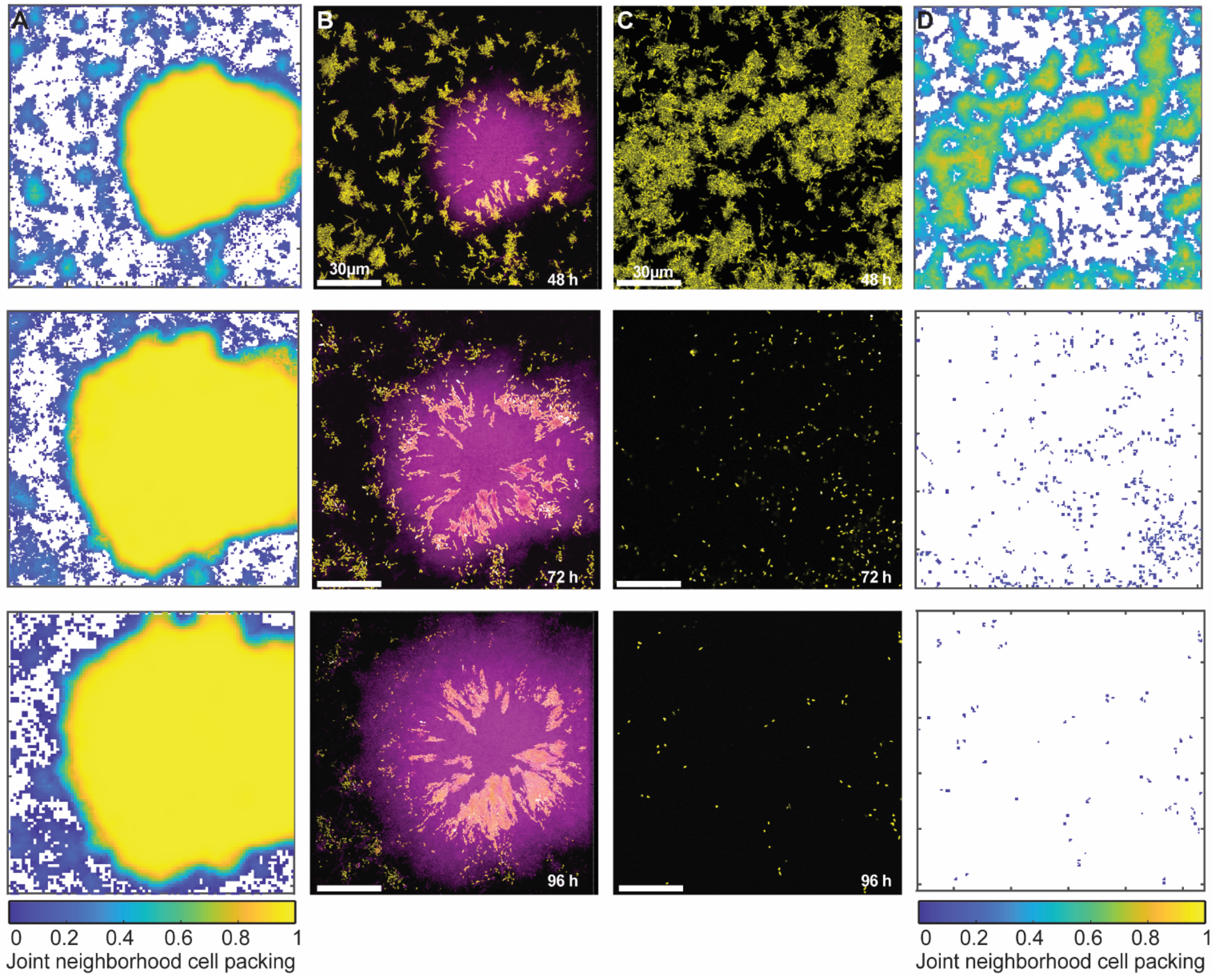

SI Figure S9. *Vibrio cholerae* biofilm architecture is maintained through time even as *E. coli* inclusions continue to grow and expand. (**A**) Heatmaps of the merged neighborhood biovolume fraction for both cell types over the time course experiment shown in (**B**). (**B**) Time course of a dual culture biofilm of *V. cholerae* (purple) and *E. coli* (yellow), with phages being introduced into this system from 48 h onward. (**C**) Time course of a monoculture biofilm of *E. coli*, with phages introduced from 48 h onward. (**D**) Heatmaps of the merged neighborhood biovolume fraction for the time course shown in (**D)**. The data underlying this figure can be found in S1 Data.
