# Supplementary material for: Multispecies biofilm architecture determines bacterial exposure to phages
Source: PLoS Biol. 2022 Dec 22;20(12):e3001913. doi: 10.1371/journal.pbio.3001913 (PMC9778933; doi:10.1371/journal.pbio.3001913)
Supplement: Attachment — Submitted filename: Winans_ReviewerResponse_2022.pdf [file pbio.3001913.s013.pdf]

Editorial and referee comments are pasted below as originally received from the editorial board at *eLife*. We include our responses and reference to lines in the revised text directly below each corresponding comment for ease of reading. Our responses are denoted in blue type.

Essential Revisions (for the authors):

The reviewers were all impressed by the thoroughness of the work and are favorable for publication if the following minor modifications are made:

1) Please amend the Methods to describe their bacterial strains, or the work could come under criticism, especially with all the attention by NIH to replicability.
We have added full descriptions of the parental strains and derivatives in line with referee requests. The Methods section is now revised to address these points in lines 512-527. We have also expanded Table 1 to include reference information for all strains, reagents, and software used in the study (beginning line 531).

2) Please be more specific about the nature of biological replication and if separate experiments were not performed, please perform them and add them to the supplement.
We have added detail to the methods section to provide full clarity on the execution of independent replicates, which for core experiments were all performed in independent flow devices, inoculated from independent starting cultures, on different days, weeks, and in most cases, months. In one case where biological replicates were performed all in one week, we repeated the experiments (now 10 months later) and added these data to the relevant figure so that these replicates spanned multiple weeks and months as well. Lines 632-638 and Figure 4.

3) See other minor details below.

----

eLife's editorial process also produces an assessment by peers designed to be posted alongside a preprint for the benefit of readers.

eLife Assessment:

The authors use high-resolution imaging to explore how a bacterial species (E. coli) can escape phage attack (by phage T7 or lambda) in biofilms formed together with a competitor species (V. cholerae). This is an important question, as that most of our knowledge on phage-bacterial interactions comes from single-species biofilms, which are not representative of natural biofilms. The paper shows unambiguously that E. coli survive phage introduction primarily when they are embedded in (and thus protected by) the phage-insensitive Vibrio. The study is well-designed and no major weaknesses were noted. The work sets a new standard in resolving phage dynamics in structured bacterial environments and likely is relevant to countless other systems, in particular to phage therapy applications involving biofilms.

We are very grateful to the referees for their careful reading of our original submission, and we were thrilled to see that their impressions of the work were so positive and encouraging. We appreciate that the referees agreed there were no major weaknesses in the original submission, but several points were noted at which we could provide further clarity for the readers, or further reassurance for the robustness of our results. We have endeavored to carefully address every point for requested clarification in the text. We have also performed some additional runs of pertinent experiments to demonstrate that all of our results replicate robustly when performed on different days, weeks, and months. Once again, we would like to thank the referees for their attentive reading and for their comments, which have helped us to further strengthen our paper.

Reviewer #1 (Public Review):

In this paper, the authors ask how the presence of competitor species can affect the infection of a focal species by phage. This is a very important question, given that most of our knowledge on phage-bacterial interactions has been inferred from single-species biofilms, although we are aware that this is not representative of natural biofilms.

The experimental system the authors have at hand is a great one to ask this question, given the ability to follow individual cells of each of the two species as well as the phage, to distinguish infected from non-infected cells and to construct mutant strains to alter biofilm architecture and phage susceptibility.

Using this toolbox, the authors have designed careful experiments to test their hypotheses, with strong evidence to support their conclusions and clear exposure to the knowledge gaps that remain to be filled in future work. In summary, they show that the tight packing of V. cholerae biofilms can limit exposure of E. coli to phage and the spread of phage if small areas of infection do occur. This protective effect occurs despite competition between the two species.

My only recommendations are to clarify whether experiments have been sufficiently replicated (whether biological replicates come from independent experiments) and a more in-depth discussion on 1. how E. coli uses curli fibers to protect itself against phages in a more ecologically relevant context (have they evolved to be expressed only when alone? What is the best moment to express them? Does it depend on who's there?) and 2. what is expected with phage infection in the presence of partners other than V. cholerae.

This work will be important reading for anyone studying biofilms and/or phage infection. It is also interesting for researchers thinking about between-species interactions and the subtlety of how interactions can play out as layers of beneficial and detrimental effects of one species on another.

We would like to reiterate our thanks to the first referee for their careful assessment of our work and helpful suggestions for clarification of key points regarding replication design and discussion of the broader ecological contexts for our results. The note on replication is repeated below, and we address it there in detail. Regarding the discussion points (1) and (2) noted by the referee above, we appreciated these suggestions and have nearly doubled the length of the Discussion with more detail regarding these points and those of the other referees noted below. A main revision to our discussion was to point out the limitations of our model system despite the crucial core principles it demonstrates; part of this revised discussion includes the importance of future work on how *E. coli* interacts with phages in different environments, including in different multispecies community contexts. We think one of the main strengths of our paper as it stands is that it makes very clear how important it will be for the research community to direct its attention to the cellular resolution details of biofilm-phage interaction for other model species and environmental conditions. The discussion includes revised and new text in lines 456-483 addressing these important questions.

Reviewer #1 (Recommendations for the authors):

As mentioned in the public review, it would be good to clarify whether any separate experiments were performed (biological replicates) or just different channels in the same experiment. If only one experiment was conducted for each data set, then I would recommend repeating the key experiments and including the data in the supplement.

For our original submission, replicates of our experiments in Figures 1-3 and the supplemental information had all been performed on multiple independent microfluidic devices inoculated from independent overnight starting cultures on different days and weeks. The experiments in Figure 4 had been performed the span of 1 week across different microfluidic devices from different overnight starting cultures. While this affords replication controlling for variance between different flow devices and overnight cultures, it did not provide control for variance across runs occurring on different week-long runs. So, we have repeated this set of Figure 4 experiments, now roughly 10 months after the original runs of this experiment, and we have incorporated the new data in the relevant panels of Figure 4. In all respects we hope to have established that our results are highly robust to replication accounting for key potential sources of variance. Please see the Methods section beginning line 531.

Apart from that, I only have minor comments:

L. 100: what culture conditions?
This point has now been clarified in the revised text with additional citations. Line 111.

L. 103: Biofilms have not produced sufficient curli matrix? According to what? Fig.? Ref?

For the microfluidic conditions used here, monoculture biofilms of *E. coli* begin to produce curli matrix approximately 55-60 hours after the start of nutrient flow in the system; this has been established by several prior papers using the same microfluidic devices and flow regimes that we use for the present study. We had omitted a citation for this statement, though, and have corrected this in the revised paper. Lines 116-117

L. 163ff: "However, we note another important difference between the E. coli that survive within V. cholerae colonies and those that survive in E. coli monoculture:
the former cells maintain positive net growth within V. cholerae colonies and recover from the initial165 population decline, whereas the latter does not. We elaborate on this point with a longer time-scale experiments in the last Results section."?
There is an important distinction between the surviving populations of early-stage *E. coli* monoculture biofilms, and the surviving populations of *E. coli* within co-culture biofilms with *V. cholerae*. Initially, these two surviving population sizes are similar, but over time, their trajectories become different. We wanted to point out that this nuance will be clarified later in the paper, given that in Figure 1, only the shorter-term survival dynamics are shown, which may prompt reader curiosity as to what happens on longer time scales. We feel the results are best communicated if we turn our attention to the mechanistic questions addressed in Figures 2 and 3, and then return to the long-term population dynamics of *E. coli* and *V. cholerae* in mono-culture versus co-culture, with or without phage exposure, which together are the focus of Figure 4. Having noted this, at the end of our discussion of Figure 1, we could have been clearer with our meaning and transitioning to the next section, which we have improved in the revised version. Lines 190-195.

L. 213: Fig. 2E (typo)
Corrected.

L. 330: Fig. 4E (typo)
Corrected.

Reviewer #2 (Public Review):

This work is an attempt to explain how bacteria may escape phage attack in biofilms. The system uses 2 bacterial species (E. coli, Vibrio cholerae) and one phage (T7 or lambda) that infects only the E. coli. With a combination of fluorescent labeling of the bacteria and high-resolution imaging, the paper shows unambiguously that E. coli survive phage introduction primarily when they are embedded in (and thus protected by) the phage-insensitive Vibrio. Furthermore, this effect is due to at least one protein secreted by Vibrio, even though the Vibrio is not binding the phage per se. This work sets a new standard in resolving phage dynamics in structured bacterial environments and likely is relevant - in some fashion - to countless other systems. It may also portend the specific nature of challenges to phage therapy applications involving biofilms.

Reviewer #2 (Recommendations for the authors):

I don't have a lot to say about this study. The results seem clear and without a doubt to the interpretation. Although the system itself is artificial (biofilms of E. coli and Vibrio and phage T7), it is easy to imagine that the findings here will generalize widely. My comments are minor.

We are grateful for the sentiments expressed by the referee, particularly with regard to their confidence in the broad relevance of our results to other species and systems. The minor points below were also very helpful in allowing us to further increase the clarity of our communication of our results' context, and to better point limitations of our work that in turn emphasize crucial directions for future work in the biofilm and phage research communities.

The paper never directly acknowledges the possible artificiality of the system (even if I think the artificiality is inconsequential to the big picture).

This point is well taken. We intended to allude to limitations of ecological realism in our experimental system in the early passages of the Results and concluding passages of our Discussion sections, but this could be done more explicitly. We

have made extensive edits to the Discussion in particular to note the limitations of our experimental system, even though, as the referee notes, these limitations do not themselves limit the scope of implications of our results. The new text for these points are in the Discussion section. Lines 456-483.

a) Is an E coli/ Vibrio biofilm thought to be common in nature?

There is documentation of co-occurring infections of *E. coli* and pathogenic *V. cholerae*, though our microfluidic biofilm growth conditions are a better approximation of microbial growth on submerged abiotic surfaces. Both species have been isolated from fouling biofilms on the hulls of marine vessels in proximity to human populations. So there is some evidence that these two species may co-occur, perhaps not uncommonly, especially near people. That is not to say that they are frequent biofilm growth partners ubiquitously in the environment. However, it is not unreasonable to propose that they encounter each other, and regardless of the frequency at which they do so, we can say for certain that *E. coli* and *V. cholerae* represent two fundamentally distinct sets of mechanisms for surface attachment, matrix production, and subsequent biofilm architecture. As such, and given their unique tractability and history of study, they are ideal tools for exploring the core principle of how phage-host interaction can change in a multispecies biofilm context in comparison with mono-species biofilm contexts. These strengths, rather than exact ecological realism, are the main reasons we chose to use them for this work, and we would agree with the referee here that any limitations in ecological realism are vastly out-weighed by the clarity that these species offer in experimental interpretation. These strengths allowed us to the overall points of our paper, and we agree with the referee that these overall lessons will apply broadly to other species combinations, and perhaps even more importantly, highlight that studying other sets of species and aiming to increase biofilm ecological realism even further are essential directions for future work for both biofilm and phage biologists. As noted above, these points can all be elaborated more clearly than they were in the originally submitted text, which we have made a concerted effort to correct in our revised paper. See in particular lines 458-467.

b) The E coli strain AR3110 looks to be a K-12 version of W3110, but I couldn't find the actual designation in the convoluted maze of references going back in time (Serra et al. indicate that all strains are derivatives of W3110 but I don't see AR3110 in that paper, which is supposedly the source). And the Methods section on Bacterial strains doesn't reference Table 1. Why do I care? If a K-12 strain is indeed used here, then it should be pointed out that T7 does not infect the true wild-type E. coli, which would have its O-antigen intact. So, again, the system is artificial. I doubt it matters, but it should be acknowledged. The antigenic characteristics of the E. coli and Vibrio should be given in this paper (and Table 1 should indicate which strains were used in what assays).

We appreciate the referee's attention to detail here, and we have updated the strain Table and the methods section with the pertinent information with regard to AR3110. We can confirm that this strain is a derivative of the K-12 strain W3110 produce by Regine Hengge's laboratory. W3110 has a stop codon in its *bcsQ* locus that results in complete inactivation of its cellulose operon. This *bcsQ* allele has been replaced with a functional copy in AR3110 to produce a strain with restored cellulose production (which is an *E. coli* biofilm matrix component), but which is otherwise isogenic to W3110. Per the referee's suggestion, we have made a note of this with updated references to direct readers to the paper in which this strain was introduced, as well as a note regarding O-antigen variation among known *E. coli* isolates and the importance of this detail for susceptibility to T7 phage.

As a derivative of K-12, AR3110 does not produce an intact O-antigen, and so is sensitive to T7 phages; while this means that T7 may not affect many environmental strains of *E. coli* (which do generally have O-antigen), we note that we also showed that our core result applies equivalently to phage Lambda (SI Figure S3), which attaches to the LamB maltose importer regardless of O-antigen status. So, as also noted by the referee here, we do not think these details detract from the overall importance of our results. We have added additional strain detail in the revised Methods section lines 513-522 and Table 1.

The paragraph beginning line 205 described what I considered to be superfluous results - T7 infection in a mixed liquid culture. Indeed, the following paragraph even acknowledges this point. All the liquid culture convincingly shows is that Vibrio (in liquid) is not irreversibly binding T7.

We appreciate the referee's note here. While the experiments documenting that T7 does not bind to the *V. cholerae* cell surface are perhaps more important (Figure 2E), we feel that the liquid co-culture data (Figure S1D) control for an additional different factor, namely the possibility that *E. coli* physiology changes in co-culture with *V. cholerae*

(independently of the biofilm context) in a manner that might influence *E. coli* susceptibility to T7 binding and infection. The Figure S1D experiments refute this possibility; the figure 2E data do not refute this possibility. Though this possibility was not very likely to be true in light of the other results reported here, we still feel it would be appropriate to retain these experiments in the revised version of the paper for the sake of rigor and completeness.

This example fits into a larger picture of what has now been called 'phenotypic flux.' The process is one in which bacteria spit out a variety of phenotypes that are somewhat heritable (usually not mutations in the usual sense). The environment (in this case phages) show up and wipe out sensitive states. The survivors persist and multiply, perhaps with some continuing erosion due to the environment. It was perhaps first described for cell-cell variation in receptor abundance. Here, the 'variation' happens to be where the cell lands and starts growing. The case here is unique and interesting, but it may help readers appreciate the example if it's put in this larger context. The review/synthesis of this phenomenon is here: https://academic.oup.com/ve/advance-article/doi/10.1093/ve/veac086/6701588.

We appreciate the referee's bringing this review paper and perspective to our attention. The idea of phenotypic flux maintaining coexistence between susceptible bacteria and their phages is an interesting one. This general idea is that *E. coli* hosts exhibit variance on a population scale in elements of metabolic, transcriptional/translational, and cell surface physiology, not due to heritable differences between mutants but rather because of, for example, noise in gene expression or variability in cells' cell cycle state. Some of these physiological states may be less susceptible than others, or not susceptible at all, to phage infection. The presence of phages may then shift the physiological composition of the population such that a higher fraction exists in the physiological states that are less phage susceptible than would be the case in the absence of phage exposure, which can in turn enable phage-host coexistence.

This is an interesting conceptual framework, though we are not sure of the extent to which our example applies: in our study, *E. coli* in biofilm co-culture with *V. cholerae* happen to survive phage exposure not because of population variance in *E. coli* physiology, but because of random chance as to where they happen to colonize the surface during colonization and whether they were close enough to *V. cholerae* to become embedded in a *V. cholerae* cell group prior to the introduction of phages. The determining factors here are most likely the relative proportions of *E. coli* and *V. cholerae*, the density with which the bottom surface is initially colonized by the two species, and the amount of time of biofilm growth prior to the introduction of phages. While we're not sure if this falls under the umbrella of the phenotypic flux idea, it still did not receive quite enough attention in the Discussion section, so we have added text for these points in the revised addition, also adding a reference to this paper for readers interesting in exploring this potential connection. Lines 445-455.

Reviewer #3 (Public Review):

In this study, the authors explored if the microscale biofilm structure of a model two-species biofilm (E. coli and V. cholerae) impacted cell-cell and cell-phage interactions. To do so, the authors grew biofilms and imaged them using fluorescence microscopy. Then they quantitively analyzed the results to determine the relative abundance of each bacterial species, interspecies proximity, and intraspecies cell-cell packing.

By comparing mono-culture E. coli biofilms to biofilms of E. coli co-cultured with V. cholerae, the authors observed that E. coli experiences a growth disadvantage when co-cultured with V. cholerae. Additionally, the authors tested whether co-culturing E. coli with V. cholerae impacts curli production. Since this experiment was performed using biofilms cultured in microfluidic flow devices, a decrease in curli protein during co-culture could have been due to V. cholerae producing a factor that decreased curli polymerization or bacterial retention of curli, since non-polymerized/non-bacterially-retained curli could wash out of the flow device and not be detected. As such, it was important that the authors tested both the transcription of the csgBAC operon and curli expression. In these experiments, the authors observed that co-culturing E. coli biofilms with V. cholerae decreased curli production, and although they did not uncover a specific mechanism for this, they were able to determine that it was due, at least in part, to a decrease in transcription of the csgBAC operon. It would be interesting to see if curli levels were impacted in co-culture in other biofilm model systems, such as in macro-colony biofilms. Overall, the results of these experiments support the authors' conclusion that in the absence of phages, the two species compete for limited space and resources, and E. coli is hindered in its ability to make curli, which in mono-culture is important for phage protection.

The authors tested if despite that co-culture decreased E. coli biofilm fitness in the absence of phage, E. coli was protected

from T7 phage during co-culture with V. cholerae. To test this, early growth stage biofilms (45 min) were exposed to T7 phages, and the impact on E. coli abundance and localization was determined. The authors quantitatively analyzed their microscopy images, and they found that E. coli that survived T7 phages were contained in clusters that were mostly V. cholerae and had high cell packing density. They found that with pre-phage exposure, there is less total E. coli in the co-cultured biofilm flow cell. However, of the starting E. coli, a higher percentage survive phage exposure in the co-culture. Additionally, by testing a curli-null strain, they found that co-culture allowed E. coli to survive phage exposure without having to elaborate a curli-based matrix. Overall, the results demonstrated that E. coli that was embedded in tightly packed clusters of V. cholerae were protected from phage.

A previous study supported that tight cell-cell packing in V. cholerae biofilms protected those bacteria from phage. Based on this past finding, and the observation that only E. coli that were embedded in tightly packed V. cholerae aggregates were protected from T7 phage, the authors hypothesized that biofilm architectural features drove the observed protection of E. coli from T7 phage when the two bacterial species were co-cultured. To test their hypothesis, the authors performed tested whether E. coli was protected from T7 phage when it was co-cultured with a V. cholerae strain that was not able to pack tightly (ΔrbmA). They confirmed that indeed the ΔrbmA strain was not as tightly packed as WT V. cholerae, and they also observed that E. coli was not protected from T7 phage when it was co-cultured with this strain. Overall, the results of this experiment supported the authors' conclusion that it is biofilm architectural features (tight cell-cell packing) that was protective.

In summary, this was a well-designed study and no major weaknesses were noted. The results supported the authors' conclusion that E. coli experiences a growth disadvantage when co-cultured with V. cholerae, but that E. coli that is embedded within tightly structured V. cholerae biofilm aggregates is more protected from T7 phage independently of curli production. The major strength of this study was that by using fluorescence microscopy to assess bacterial abundance, the authors were able to discern the impact of biofilm architecture and microscale biogeography on the impact of co-culture on protection from phage, which is something that cannot be determined using a "bulk" approach like determining CFUs.

In general, past studies of biofilms have focused on mono-species biofilms, and additionally have lacked insight into the effects of biofilm architecture. The findings of this study and the method used in the study provide a way of moving towards studying more biologically relevant systems since bacteria often exist as multi-species communities in the environment and in human infection. Additionally, with some recent pushes to develop phage therapy as an antibacterial therapeutic, it is crucial that we gain an understanding of how co-culture may impact susceptibility to phage. For example, prior to this study, it would have been assumed that E. coli that is deficient in curli production would be susceptible to phage. However, as this study showed, co-culture can dramatically change this view.

We are very grateful to the referee for their highly detailed reading of our manuscript and impressions of our work.

Reviewer #3 (Recommendations for the authors):

The wording in the paragraph that starts at 155 is a bit confusing (I had to read it a few times). To improve its clarity, you might consider re-wording it or adding a schematic.

We agree that this paragraph could be delivered more fluently to convey the intended messages clearly to readers, and also to anticipate the experiments that will follow in the later stages of the paper and Figure 4. This point is related to one raised by referee #1 as well, and we have made modifications to the text in this paragraph for better clarity. Lines 190-195.

Small note: missing color key for Figure 4 to indicate that E. coli is yellow and V. cholerae is magenta.

We appreciate the close attention to details on the part of the referee. This has now been corrected.
