# Supplementary material for: Multispecies biofilm architecture determines bacterial exposure to phages
Source: PLoS Biol. 2022 Dec 22;20(12):e3001913. doi: 10.1371/journal.pbio.3001913 (PMC9778933; doi:10.1371/journal.pbio.3001913)
Supplement: Attachment — Submitted filename: Winans_ResponseToComments_2.pdf [file pbio.3001913.s015.pdf]

Response to Editorial and Review Comments:

Editorial comments are pasted below as originally received in correspondence from the *PLoS Biology* office. **Our responses are placed directly below each corresponding comment in blue text for clarity.**

Thank you for your patience while we considered your revised manuscript "Cell group architecture dictates phage exposure in multispecies biofilms" for publication as a Research Article at PLOS Biology. This revised version of your manuscript has been evaluated by the PLOS Biology editors and the Academic Editor.

Based on our Academic Editor's assessment of your revision, we are likely to accept this manuscript for publication, provided you satisfactorily address the following data and other policy-related requests.

IMPORTANT: Please attend to the following:

a) Please change the title to something more explicit; we suggest something like "Cellular architecture of multispecies biofilms determines phage exposure" or even "Cellular architecture of multispecies biofilms determines each host species' exposure to phage" (I note that we found the phrase "Cell group architecture" somewhat opaque)

To improve readability while also keeping emphasis on the arrangement of cell groups in space, we would like to suggest the following new title: **"Multispecies biofilm architecture determines bacterial exposure to phages".** We have made this update to the revised manuscript and the metadata entries for our paper in the PLoS Biology author portal.

b) The Academic Editor was concerned that the graphs in Fig 4 didn't seem to change visibly during revision, despite the new data. To my eye, the graphs seem subtly different, and I see that the sample numbers have changed in the legend; however, the Academic Editor requested that you "either highlight the repetition and the subsequent changes to figure 4 or include a supplemental figure showing evidence of this repetition."

We appreciate the editors' question here and would like to make note that only a sub-section of the data in Figure 4, panel E, had not already been replicated across multiple days and weeks in the original submission of the first version of this manuscript. Specifically, only the grey square and grey circle *E. coli/V. cholerae* co-culture experiments (with and without phages) needed to be repeated in response to this original comment. Therefore, when additional replication was performed, the resulting changes to the figure were subtle, because only the grey data in Figure 4E changed relative to the original submission. We have included below a comparison of the original version of Figure 4E and the updated version with the new data incorporated, and hope this provides clarity on the incorporation of the additional experimental replicates. We have highlighted with the transparent bar that the grey data trajectories changed relative to the original submission, although not in substantial way and without altering the overall pattern we reported.

Original submission

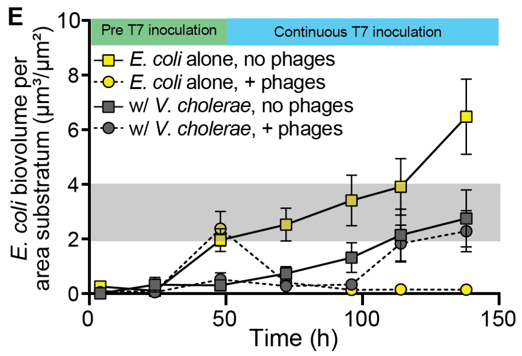

Revised submission

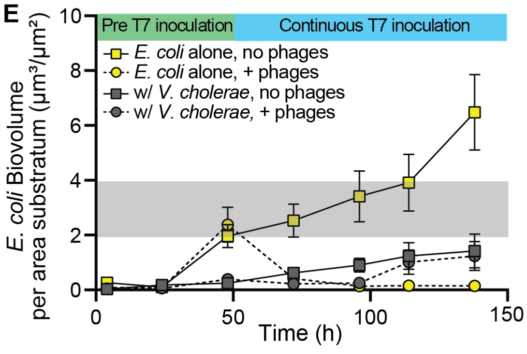

c) The Academic Editor also asked that you include a schematic or Figure of the initial microfluidic device used in this study as a supplementary Figure. ("With this growing field, I think it is important to properly document these devices as there is a lot of variation in their shape and design that impacts the reported results.")

We agree with the editor and have now included a schematic outline of the microfluidic device design, along with relevant chamber dimensions, that was used for the experiments in this paper. This is now included as a new supplementary figure (SI Figure S10) that is referenced in the methods section. A copy of this diagram and its figure legend are provided below.

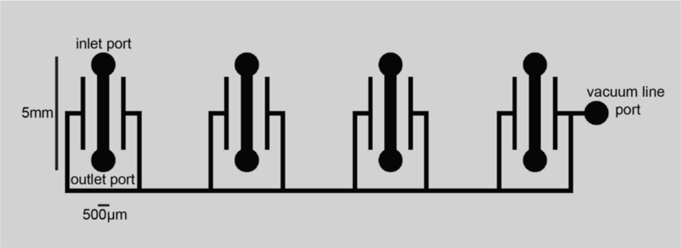

SI Figure S10. A diagram of the microfluidic device chamber design used for all biofilm growth experiments in this study. This example contains 4 parallel chambers, each with an inlet and an outlet port for connection to fluid inlet/outlet tubing. Technical replicate image stacks were taken from within the straight rectangular section between the rounded inlet and outlet ports of a chamber. The thinner, continuous vacuum line surrounding the 4 separated chambers was connected to a wall vacuum line to apply negative pressure; this method discourages the introduction of air bubbles into the liquid-filled portions of the flow chambers by drawing any small bubbles from flow chambers through the PDMS into the surrounding air-filled vacuum line.

d) Many thanks for your very thorough data provision. Please could you cite the location of the data clearly in all relevant main and supplementary Figure legends, e.g. "The data underlying this Figure can be found in S1 Data."

We have added the requested reference to the S1 Data file to each of the relevant main and SI figures as requested.

As you address these items, please take this last chance to review your reference list to ensure that it is complete and correct. If you have cited papers that have been retracted, please include the rationale for doing so in the manuscript text, or remove these references and replace them with relevant current references. Any changes to the reference list should be mentioned in the cover letter that accompanies your revised manuscript.
